# DNA Data Bank of Japan: 30th anniversary

**DOI:** 10.1093/nar/gkx926

**Published:** 2017-10-10

**Authors:** Yuichi Kodama, Jun Mashima, Takehide Kosuge, Eli Kaminuma, Osamu Ogasawara, Kousaku Okubo, Yasukazu Nakamura, Toshihisa Takagi

**Affiliations:** DDBJ Center, National Institute of Genetics, Shizuoka 411–8540, Japan; National Bioscience Database Center, Japan Science and Technology Agency, Tokyo 102–8666, Japan

## Abstract

The DNA Data Bank of Japan (DDBJ) Center (http://www.ddbj.nig.ac.jp) has been providing public data services for 30 years since 1987. We are collecting nucleotide sequence data and associated biological information from researchers as a member of the International Nucleotide Sequence Database Collaboration (INSDC), in collaboration with the US National Center for Biotechnology Information and the European Bioinformatics Institute. The DDBJ Center also services the Japanese Genotype-phenotype Archive (JGA) with the National Bioscience Database Center to collect genotype and phenotype data of human individuals. Here, we outline our database activities for INSDC and JGA over the past year, and introduce submission, retrieval and analysis services running on our supercomputer system and their recent developments. Furthermore, we highlight our responses to the amended Japanese rules for the protection of personal information and the launch of the DDBJ Group Cloud service for sharing pre-publication data among research groups.

## INTRODUCTION

The DNA Data Bank of Japan (DDBJ, http://www.ddbj.nig.ac.jp) (1) is a public database of nucleotide sequences established at the National Institute of Genetics (NIG, https://www.nig.ac.jp/nig). Since 1987, the DDBJ has been collecting annotated nucleotide sequences as its traditional database service and we held the NIG international symposium commemorating its 30th anniversary in May 2017 (http://www.ddbj.nig.ac.jp/ddbj30th/en). The content of the DDBJ is primarily accumulated via submissions of sequence data by researchers. In addition, the Japan Patent Office and the Korean Intellectual Property Office also contribute sequences from published patent applications. This endeavor has been conducted in collaboration with GenBank (2) at the National Center for Biotechnology Information (NCBI) and with the European Nucleotide Archive (ENA) (3) at the European Bioinformatics Institute (EBI). The collaborative framework is called the International Nucleotide Sequence Database Collaboration (INSDC) (4) and the product database from this framework is called the International Nucleotide Sequence Database (INSD).

Within the INSDC framework, the DDBJ Center also services the DDBJ Sequence Read Archive (DRA) for raw sequencing data and alignment information from high-throughput sequencing platforms (5), BioProject for sequencing project metadata and BioSample for sample information (1,6). The comprehensive resource of nucleotide sequences and associated biological information complies with the INSDC policy that guarantees free and unrestricted access to data archives (7).

In addition to these unrestricted-access databases, the DDBJ Center services a controlled-access database, the Japanese Genotype-phenotype Archive (JGA, http://trace.ddbj.nig.ac.jp/jga), in collaboration with the National Bioscience Database Center (NBDC, https://biosciencedbc.jp/en) of the Japan Science and Technology Agency (1, 8). The JGA stores genotype and phenotype data from individuals who have signed consent agreements authorizing data use only for specific research. The data access is strictly controlled, similar to the data access policy of the database of Genotypes and Phenotypes at the NCBI (9,10) and the European Genome-phenome Archive at the EBI (11). NBDC provides the guidelines and policies for sharing human-derived data (https://humandbs.biosciencedbc.jp/en/guidelines) and also reviews data submission and usage requests.

The DDBJ Center, a part of NIG, is funded as a supercomputing center. Our web services, including submission systems, data retrieval and analytical systems and backend databases, are performed on the NIG supercomputer system. The current commodity-based cluster was implemented in 2012 (12).

In the present article, we report the update of the above services at the DDBJ Center, highlight our responses to the amended Japanese rules for protection of personal information and describe the launch of the DDBJ Group Cloud (DGC) service for sharing pre-publication data among research groups. All resources described here are available at http://www.ddbj.nig.ac.jp and most of the archival data can be downloaded at ftp://ftp.ddbj.nig.ac.jp.

## DDBJ ARCHIVAL DATABASES

### Data contents: traditional DDBJ and the DDBJ sequence read archive

In 2016, most of the nucleotide data submissions to the DDBJ were made by Japanese research groups (3750 times; 73.7%), with the rest coming from Thailand (198 times; 3.9%), Iran (186 times; 3.7%), Egypt (176 times; 3.5%), South Korea (168 times; 3.3%), China (151 times; 3.0%) and other countries and regions (462 times; 9.1%).

From this report, DDBJ periodical release includes not only conventional sequence data but also bulk sequence data, such as Whole Genome Shotgun (WGS) and Transcriptome Shotgun Assembly (TSA). Between June 2016 and May 2017, the DDBJ periodical release increased by 147 437 521 to 874 923 909 in terms of the number of entries and by 572 071 571 206 to 2 461 362 329 556 in terms of the number of base pairs. The periodical release does not include third party data (TPA) records (13). The DDBJ contributed 7.23% of the entries and 3.79% of the total base pairs in the nucleotide sequence data of INSD. A detailed statistical breakdown of the number of records is shown on the DDBJ website (http://www.ddbj.nig.ac.jp/breakdown_stats/prop_ent-e.html). Noteworthy large-scale data released from DDBJ are listed in Table 1.

**Table 1.** List of large-scale data released by the DDBJ sequence databases from June 2016 to May 2017

Data type	Organism	Accession numbers for annotated sequences (number of entries)	Accession numbers for reads (submission number)
Genome	Japanese quail, *Coturnix japonica*	WGS: BASJ02000001-BASJ02009499 (9499 entries)	DRR002288-DRR002301 (DRA000595)
			DRR055128-DRR055129 (DRA004460)
	Japanese white stork (*Ciconia boyciana*)	BDFF01000001-BDFF01505419 (505 419 entries)	n/a
	red-crowned crane (*Grus japonensis*)	WGS: BDFG01000001-BDFG01357545 (357 545 entries)	n/a
	Okinawa rail (*Gallirallus okinawae*)	WGS: BDFH01000001-BDFH01768680 (768 680 entries)	n/a
	red algal species (*Liagora japonica*)	WGS: BCQK01000001-BCQK01275014 (275 014 entries)	DRR041863 (DRA003813)
		WGS: BCQL01000001-BCQL01381344 (381 344 entries)	DRR041864 (DRA003813)
	sub clover, *Trifolium subterraneum* cv. Daliak	CON: DF973112-DF976994 (3883 entries)	DRR018263-DRR018264 (DRA002213)
		WGS: BCLP01000001-BCLP01066167 (66 167 entries)	DRR032035-DRR032043 (DRA003274)
	sub clover, *Trifolium subterraneum* cv. Woogenellup	WGS: BBPR01000001-BBPR01968279 (968 279 entries)	DRR018261-DRR018262 (DRA002213)
	water bear, *Ramazzottius varieornatus*	GSS: FT955276-FT997721 (42 446 entries)	n/a
		WGS: BDGG01000001-BDGG01000199 (199 entries)	DRR013908-DRR013910 (DRA001119)
		fosmid clones: AP013349-AP013352 (4 entries)	
		mitochondrion: AP017609 (1 entry)	
	quinoa, *Chenopodium quinoa*	WGS: BDCQ01000001-BDCQ01024845 (24 845 entries)	DRR057247-DRR057301 (DRA004558)
	pink oyster mushroom (*Pleurotus salmoneostramineus*)	WGS: BDGN01000001-BDGN01026934 (26 934 entries)	
	Okinawa mozuku, *Cladosiphon okamuranus*	CON: DF977685-DF978416 (732 entries)	DRR059718-DRR059726 (DRA004654)
		WGS: BDDF01000001-BDDF01004525 (4525 entries)	
	Japanese morning glory (*Ipomoea nil*)	WGS: BDFN01000001-BDFN01003416 (3416 entries)	DRR013917-DRR013926 (DRA001121)
		mitochondrion: AP017303 (1 entry)	DRR024668 (DRA002710)
		chloroplast: AP017304 (1 entry)	DRR048755-DRR048757 (DRA004158)
		GSS: GA933005-GA974698 (41 694 entries)	n/a
		n/a	RAD-Seq: DRR026831-DRR027252 (DRA002758)
	crown-of-thorns starfish, *Acanthaster planci*	CON: DF978489-DF980253 (1765 entries)	DRR064078-DRR064083 (DRA004863)
		WGS: BDGF01000001-BDGF01018088 (18 088 entries)	
		CON: DF980254-DF983527 (3,274 entries)	DRR064073-DRR064077 (DRA004862)
		WGS: BDGH01000001-BDGH01019917 (19 917 entries)	
	bitter gourd, *Momordica charantia*	WGS: BDCS01000001-BDCS01001052 (1052 entries)	DRR056762 (DRA004516)
			DRR057118-DRR057122 (DRA004548)
	Para rubber tree, *Hevea brasiliensis*	WGS: BDHL01000001-BDHL01592579 (592 579 entries)	n/a
	common fig, *Ficus carica*	WGS: BDEM01000001-BDEM01027995 (27 995 entries)	n/a
	shiitake mushroom, *Lentinula edodes*	WGS: BDGU01000001-BDGU01001951 (1951 entries)	n/a
transcriptome	cherry salmon, *Oncorhynchus masou masou*	TSA: IABA01000001-IABA01097925 (97 925 entries)	DRR065944, DRR065945 (DRA004887)
	water bear, *Ramazzottius varieornatus*	EST: HY377478-HY448296 (70 819 entries)	n/a
	Chinese lantern (*Physalis alkekengi* var. franchetii)	TSA: IABG01000001-IABG01075221 (75 221 entries)	DRR048294-DRR048297 (DRA004085)
	cape gooseberry (*Physalis peruviana*)	TSA: IABH01000001-IABH01054513 (54 513 entries)	DRR048298-DRR048300 (DRA004085)
	jellyfish, *Turritopsis* sp. SK-2016	TSA: IAAF01000001-IAAF01090327 (90 327 entries)	DRR053671-DRR053676 (DRA004346)
	Japanese morning glory (*Ipomoea nil*)	EST: HY917605-HY949060 (31 456 entries)	n/a
		n/a	DRR024544-DRR024549 (DRA002647)
	Yamato shrimp (*Caridina multidentata*)	TSA: IABX01000001-IABX01137038 (137 038 entries)	DRR054560-DRR054562 (DRA004369)
	sea slater (*Ligia exotica*)	TSA: IABZ01000001-IABZ01111125 (111 125 entries)	DRR054553-DRR054554 (DRA004368)
	common house spider (*Parasteatoda tepidariorum*)	TSA: IABY01000001-IABY01023144 (23 144 entries)	DRR054577 (DRA004377)
		TSA: IACA01000001-IACA01110557 (110 557 entries)	DRR054572-DRR054576 (DRA004370)
	Japanese cedar (*Cryptomeria japonica*)	TSA: FX334350-FX347193 (12 844 entries)	DRR001824-DRR001831 (DRA000521)
		HTC (full length insert cDNA):	n/a
		AK406520-AK407765, AK407767-AK410326, AK410328-AK410473,	
		AK410475-AK410519, AK410521-AK410553, AK410555-AK410823,	
		AK410825-AK411144, AK411146-AK411166, AK411168-AK411382,	
		AK411384-AK411486, AK411488-AK411994, AK411996-AK412150,	
		AK412152-AK412174, AK412176-AK412481, AK412483-AK413809,	
		AK413811-AK414131, AK414133-AK414473, AK414475-AK415106,	
		AK415108-AK416748, AK416865-AK416866 (10 213 entries)	
		EST: FY225484-FY260943, FY261885-FY298838,	
		FY762882-FY780692 (90 225 entries)	
	ant (Diacamma sp. Okinawa-2006a)	TSA: IACE01000001-IACE01168226 (168 226 entries)	DRR024699-DRR024728 (DRA002714)
	1829 samples from the major human (*Homo sapiens*) primary cell types and tissues.	n/a	DRR063026-DRR063057 (DRA004812)
			DRR063058-DRR063070 (DRA004813)
			DRR063071-DRR063083 (DRA004814)

In the period between June 2016 and May 2017, high-throughput sequencing data of 30 418 runs were registered to the DRA. Some of the RIKEN FANTOM5 transcript data (58 runs in total) used to generate a comprehensive atlas of 27 919 human long non-coding RNA genes and expression profiles across 1,829 samples from the major human primary cell types and tissues (14) were released from the DRA (Table 1).

### Data contents: the Japanese genotype-phenotype archive (JGA)

The JGA is a permanent archiving service for human genotype and phenotype data (8). Submitters must remove any direct personal identifiers from metadata to be submitted to the JGA. After encrypting the submitted data, the JGA team stores them in the secure database. As of 17 August, 2017, the JGA had archived 104 studies (81 TB) of individual-level human datasets submitted by Japanese researchers. Submission of these studies was reviewed and approved by the Data Access Committee (DAC) at the NBDC. The summaries of 57 studies are available to the public on both the JGA (https://ddbj.nig.ac.jp/jga/viewer/view/studies) and the NBDC (https://humandbs.biosciencedbc.jp/en/data-use/all-researches) websites. Notable studies available for data access request include ‘Standard epigenome mapping in human epithelial cells of the digestive and urogenital organs’ (JGA study accession numbers JGAS00000000078–80) submitted by the Japanese team of the International Human Epigenome Consortium (http://crest-ihec.jp/english/index.html) and ‘GWAS for atrial fibrillation in the Japanese population’ (JGAS00000000114), which is part of the BioBank Japan project that conducted genome-wide association analyses of over 200 000 Japanese participants related to 47 common diseases (15). To access individual-level data of these public studies, users are required to make data access requests to the NBDC (https://humandbs.biosciencedbc.jp/en/data-use). The DAC at the NBDC ensures that the stated research purposes are compatible with participant consent and that the principal investigator and institution will abide by the NBDC guidelines and the specific terms and conditions imposed for a given dataset. Once access has been granted by the DAC, datasets with access permission can be downloaded with a secure software tool provided by the JGA. It is necessary for users to establish a secure computing facility for local use of the downloaded data according to the NBDC security guidelines.

### Responses to the amended rules for protection of personal information

The DDBJ Center handles personal information in compliance with Japanese laws and guidelines. The Act on the Protection of Personal Information (PPI Act, https://www.ppc.go.jp/en/legal) first established in 2003 defines the categories of personal information that should be protected and how this should be achieved. Reflecting information and communication technology developments that have markedly increased the nature and usage of personal information, the PPI Act was amended. The following two amendments have had a major influence on the sharing of personal genotype and phenotype information. (i) Personal whole-genome-level DNA sequence data are defined as ‘individual identification code.’ Even if all personal identifiers have been removed from the metadata linked to the whole-genome-level DNA sequencing data, these data need to be handled as ‘personal information’ because the DNA sequences are inherently a code that could identify individuals. (2) Personal information including the individual's race and medical history, which require special consideration so as not to cause unfair discrimination or prejudice against the individual, is defined as ‘sensitive personal information.’ To acquire sensitive personal information and provide it to others, researchers are in principle required to obtain informed consent from research participants. In accordance with the PPI Act amendment, the relevant ministries’ ethical guidelines for medical and health research involving human subjects have also been amended. After the enforcement of these amended laws and guidelines on 30 May, 2017, to submit whole-genome-level personal genomic DNA sequencing data to our unrestricted- or controlled-access databases, the submitter needs approval from the NBDC, which checks whether the submission complies with the amended laws and guidelines.

## DDBJ SYSTEM UPDATE

### Submission services of biological data

For annotated sequence submission to the traditional DDBJ database, we provide two systems: the Nucleotide Sequence Submission System (NSSS) (16) and the Mass Submission System (MSS) (17). The NSSS is an interactive application to enter all items via a web-based form (http://www.ddbj.nig.ac.jp/sub/websub-e.html). The MSS involves a procedure to send large-scale data files directly (http://www.ddbj.nig.ac.jp/sub/mss_flow-e.html). Both systems were enhanced to comply with the new rules of feature and qualifier usages (see http://www.ddbj.nig.ac.jp/insdc/icm2016-e.html#ft).

Submitters can register metadata to BioProject, BioSample and DRA by logging in and using the web interface (https://trace.ddbj.nig.ac.jp/D-way). Human genotype and phenotype data can be submitted to the JGA by using secure upload software.

### Retrieval and analysis services of biological data

The DDBJ Center has provided the Web BLAST (18), ClustalW (19,20), vector sequences screening system VecScreen (http://ddbj.nig.ac.jp/vecscreen/vecscreen?lang=en) and Taxonomy browser TXSearch (http://ddbj.nig.ac.jp/tx_search) services, which receive requests from web interfaces. The DDBJ Center also provides the Web API for Bioinformatics (WABI) (21–23) for large-scale data analysis and the RESTful Web API service that can process requests from computer programs. The WABI service includes BLAST, VecScreen, ClustalW, MAFFT (24,25), getentry data retrieval system via accession numbers and the ARSA keyword search system for the DDBJ flat files (12). We have semantically represented the DDBJ annotated sequence records into the Resource Description Framework (RDF) in collaboration with the Database Center for Life Science (DBCLS) (1,26,27). In collaboration with EBI ArrayExpress (28), we have also mirrored the public ArrayExpress experiment, array, and Expression Atlas data to our FTP site (ftp://ftp.ddbj.nig.ac.jp/mirror_database/arrayexpress) since December 2016.

### DDBJ pipeline

The DDBJ Read Annotation Pipeline (DDBJ Pipeline, https://p.ddbj.nig.ac.jp) is a web service for annotation analysis of high-throughput DNA sequencing reads running on the NIG supercomputer (29). We provide basic analytical functions of *de novo* assembly and reference sequence alignment using a Graphical User Interface. A *de novo* assembler, Canu (30), has been added to the pipeline, which can be utilized only for long reads of Oxford Nanopore Technologies sequencers.

### The NIG supercomputer

The NIG supercomputer is composed of calculation nodes for general-purpose (554 thin nodes, each with 64 GB memory) and memory-intensive tasks including *de novo* assembly of sequencing reads (10 medium nodes, each with 2 TB of memory and one fat node with 10 TB of memory). The calculation nodes are interconnected with InfiniBand and the total peak performance of CPUs is 372 Tflops. To support massive I/O in the big-data analysis, the NIG supercomputer is equipped with 7.1 PB of the Lustre parallel distributed file system (http://www.lustre.org). The 5.5 PB MAID (Massive Array of Idle Disks) system is used for archiving large-scale sequencing data of the JGA and INSD’s Sequence Read Archive while lowering power consumption (12).

Between June 2016 and May 2017, the number of NIG supercomputer users increased from 2501 to 2951. The criteria for issuing a user login account are shown on the web page (https://sc.ddbj.nig.ac.jp/index.php/en/criteria-for-issuing-user-login-accounts). For the convenience of the users, many biological datasets (listed at https://sc.ddbj.nig.ac.jp/index.php/ja-availavle-dbs, Japanese only) and popular bioinformatics tools (listed at https://sc.ddbj.nig.ac.jp/index.php/ja-avail-oss, Japanese only) were installed in the NIG supercomputer system. Since February 2017, we have started a billing system to share costs with users who use large-volume storage and reserve the calculation nodes for new jobs. We expect that we can promote efficient use of our computer resources and increase the sustainability of our system by sharing operating costs with users (https://sc.ddbj.nig.ac.jp/index.php/billing-system, Japanese only).

### DDBJ group cloud service for sharing pre-publication data

As the sequencing technologies advance and the amount of genomic data generated grows, it becomes critical to store, analyze and share large-scale data with research collaborators efficiently. To facilitate the sharing and analysis of pre-publication data among research groups, the DDBJ Center has operated a cloud-type service DGC on the NIG supercomputer since February 2017. In the DGC databases, users can upload and share their pre-publication data with their research collaborators in the data models which are identical to those of the public databases. Upon publication, users can submit their data by simply transferring the data from the DGC database to the corresponding public one of the DDBJ Center. The DGC hosts the AMED Genome Group Sharing Database (AGD) (http://trace.ddbj.nig.ac.jp/agd/index_e.html) as the first use case. In the AGD, researchers funded by the Japan Agency for Medical Research and Development (AMED, http://www.amed.go.jp/en) upload and share their pre-publication raw personal genome sequencing data in the JGA’s data model. Because the DGC is not a fully public service, the operating costs are shared with the DGC users.

## FUTURE DIRECTION

The ever-increasing volume of personal sequencing data makes it difficult for researchers to prepare their own secure computer resources with sufficient storage and computing power and to transfer large amounts of data online from public databases. To solve these issues, the NBDC certifies qualified secure supercomputer systems as ‘Trusted Servers’ and allows users to analyze the approved JGA dataset in the Trusted Servers in addition to their own servers. The DDBJ Center will provide the secured NIG supercomputer as a Trusted Server that is connected with the JGA system by a high-speed network, so users can smoothly download the JGA dataset and analyze their own personal genomic data in the same supercomputer.

To increase the discoverability of the JGA-archived human genomes, the DDBJ Center and NBDC collaborate to provide the Global Alliance for Genomics and Health beacon web service (https://beacon-network.org) to accept queries of specific alleles on the human reference genome.

The DDBJ Center has launched the Japan Alliance for Bioscience Information portal site (http://jbioinfo.jp/index.html) in collaboration with NBDC, DBCLS and the Protein Data Bank Japan. We will develop this portal site as a one-stop service of databases and tools that are helpful in various fields of life science research.
